# Clinical impact of pre-existing acute exacerbation in patients with interstitial lung disease who underwent lung transplantation

**DOI:** 10.1186/s12931-023-02614-z

**Published:** 2023-12-07

**Authors:** Hyeon Hwa Kim, Ho Cheol Kim, Tae Sun Shim, Jee Hwan Ahn, Jin Won Huh, Sang-Bum Hong, Geun Dong Lee, Dong Kwan Kim, Seung-Il Park, Sehoon Choi

**Affiliations:** 1 Division of Pulmonology and Critical Care Medicine, Department of Internal Medicine, Asan Medical Center, University of Ulsan College of Medicine, Seoul, Republic of Korea; 2 Department of Thoracic and Cardiovascular Surgery, Asan Medical Center, University of Ulsan College of Medicine, 88 Olympic-ro 43-gil, Songpa-gu, Seoul, 05505 Republic of Korea

**Keywords:** Acute exacerbation, ILD, Lung transplantation, Mortality

## Abstract

**Background:**

Acute exacerbation of interstitial lung disease (AE-ILD) significantly impacts prognosis, leading to high mortality rates. Although lung transplantation is a life-saving treatment for selected patients with ILD, its outcomes in those presenting with AE-ILD have yielded conflicting results compared with those with stable ILD. This study aims to investigate the impact of pre-existing AE on the prognosis of ILD patients who underwent lung transplantation.

**Method:**

We conducted a single-center retrospective study by reviewing the medical records of 108 patients who underwent lung transplantation for predisposing ILD at Asan Medical Center, Seoul, South Korea, between 2008 and 2022. The primary objective was to compare the survival of patients with AE-ILD at the time of transplantation with those without AE-ILD.

**Results:**

Among the 108 patients, 52 (48.1%) experienced AE-ILD at the time of lung transplantation, and 81 (75.0%) required pre-transplant mechanical ventilation. Although the type of ILD (IPF vs. non-IPF ILD) did not affect clinical outcomes after transplantation, AE-ILD was associated with worse survival outcomes. The survival probabilities at 90 days, 1 year, and 3 years post-transplant for patients with AE-ILD were 86.5%, 73.1%, and 60.1%, respectively, while those for patients without AE-ILD were higher, at 92.9%, 83.9%, and 79.6% (p = 0.032). In the multivariable analysis, pre-existing AE was an independent prognostic factor for mortality in ILD patients who underwent lung transplantation.

**Conclusions:**

Although lung transplantation remains an effective treatment option for ILD patients with pre-existing AE, careful consideration is needed, especially in patients requiring pre-transplant mechanical respiratory support.

**Supplementary Information:**

The online version contains supplementary material available at 10.1186/s12931-023-02614-z.

## Background

The number of lung transplantations has been increasing worldwide, and interstitial lung disease (ILD) is a leading cause of lung transplantation [1]. Idiopathic pulmonary fibrosis (IPF) is the most common type of ILD, characterized by an unknown cause of irreversible and progressive pulmonary fibrosis. Over half of the patients with IPF experience a deteriorating course leading to death within 3–5 years [2, 3]. Recent studies have revealed that other ILDs, such as progressive pulmonary fibrosis (PPF), also exhibit a similar progressive course [4]. Lung transplantation is the ultimate treatment option for advanced ILD, exhibiting improved survival and quality of life [5, 6].

Acute exacerbation (AE) can occur throughout the clinical course of ILD, and refers to rapid deterioration with bilateral ground glass opacity and/or consolidation [7]. Although AE of IPF (AE-IPF) are well recognized [7, 8], AE can also occur in patients with non-IPF ILD [9, 10]. In addition, AE is usually associated with a poor prognosis and high mortality rate [10]. Although high-dose steroid therapy is commonly used in such cases per a recommendation from recent guidelines, especially in IPF patients [4], there is a lack of well-conducted randomized controlled trials to evaluate effective treatment options. Lung transplantation therefore remains the only lifesaving option with the potential for complete recovery. However, the outcomes in patients who present with pre-existing AE of ILD (AE-ILD) are conflicting compared with those of patients with stable ILD [11, 12]. Therefore, this study aimed to investigate the impact of AE on the prognosis of ILD patients who underwent lung transplantation.

## Methods

### Study population and definition

This single-center, retrospective study analyzed the data of patients with ILD who underwent bilateral lung transplantation at Asan Medical Center, Seoul, Republic of Korea, from October 2008 to January 2022. Two patients who underwent heart–lung transplantation were excluded from the study. The study period was concluded on January 31, 2022, to ensure that all patients had at least 1 year of follow-up data after the transplantation.

The revised definition of AE proposed by an international working group in 2016 in IPF [7] was applied to all ILDs, including non-IPF ILDs. The diagnostic criteria for AE included: (1) a previous or concurrent diagnosis of ILD; (2) acute worsening or development of dyspnea lasting typically less than 1 month; (3) new bilateral ground-glass opacity or consolidation superimposed on a background pattern consistent with fibrotic lung disease on computed tomography; and (4) deterioration not fully explained by cardiac failure or fluid overload. In our study, AE-ILD referred to instances in which patients were admitted because of AE and subsequently required lung transplantation due to a lack of clinical improvement. For patients with ILDs of etiologies other than IPF, PPF was characterized by the presence of at least two of the following criteria within the past year with no alternative explanation: (1) worsening respiratory symptoms; (2) physiological evidence of disease progression; (3) radiological evidence of disease progression [4].

### Perioperative management and follow-up protocol

All patients received induction immunosuppression therapy before surgery, including a high dose of intravenous methylprednisolone and basiliximab. Maintenance treatment comprised standard triple immunosuppression therapy with tacrolimus, mycophenolate mofetil, and methylprednisolone [13]. Several drugs were administered to prevent infection, including trimethoprim/sulfamethoxazole for *Pneumocystis jirovecii* for the recipient’s lifetime, ganciclovir or oral valganciclovir for cytomegalovirus for 6 months, and voriconazole for antifungal prophylaxis for 6 months.

### Outcomes and data collection

The primary outcome of this study was patient overall survival after lung transplantation. The survival duration was calculated from the date of transplantation to the date of death or the end of the study period, comprising at least 1 year of follow-up. Baseline and perioperative characteristics, including AE and postoperative outcomes, were investigated as potential risk factors for mortality. Data on demographics and clinical characteristics were extracted from the medical records. Variables included age, sex, body mass index, diseases that necessitated transplantation, pulmonary function testing, ILD-GAP index [14], pre-existing pulmonary hypertension (assessed by echocardiography or invasive right heart catheterization), smoking status, prior thoracic surgery (either lung biopsy or lobectomy), preoperative infectious episodes, use of anti-fibrotic agents, use of corticosteroids, duration of mechanical ventilation and extracorporeal membrane oxygenation (ECMO) therapy, length of intensive care unit (ICU) stay, and airway complication. Preoperative steroid usage was defined as the administration of these medications within 1 month before transplantation. It was evaluated based on the daily dose of methylprednisolone or equivalent administered within 1 month before transplantation, stratified into categories.

### Allocation system for lung transplantation in South Korea

The current allocation system for lung transplantation in South Korea is based primarily on urgency, additionally taking into account factors such as region, wait time, blood type, previous donation history, and age. Transplant candidates are stratified into categories ranging from 0 to 4, with a lower status signifying higher priority. Specifically, Status 0 is assigned to hospitalized patients reliant on mechanical ventilators and/or ECMO due to respiratory failure. In 2019, 68.6% of transplant patients in South Korea were classified as Status 0, with 38.5% undergoing ECMO as a bridge to transplantation [15]. This system differs from the Lung Allocation Score (LAS) system in the United States, which considers the benefits of transplantation [16]. Given the limitations of the lung allocation system and the shortage of donors, our institution conducted bilateral lung transplantations for all recipients.

### Statistical analysis

Continuous variables were presented as means and standard deviations, while categorical variables were presented as percentages. The comparison of continuous data was performed using Student’s *t*-test or the Mann–Whitney *U* test, while categorical data was compared using the chi-square test or Fisher’s exact test. To identify risk factors for mortality, we used Cox proportional hazards regression. The variables with a *p*-value of less than 0.2 in the univariable analysis were included in the multivariable analysis. A two-tailed *p*-value of less than 0.05 was considered statistically significant. The Kaplan–Meier method was used to plot survival curves, and the log-rank test was used to compare them. We used IBM SPSS Statistics, version 21.0 (IBM Corporation, Armonk, NY, USA) to perform the statistical analyses.

The study protocol was approved by the Institutional Review Board of the Asan Medical Center, University of Ulsan College of Medicine (IRB No. 2023 − 1175) in accordance with the guidelines of the Declaration of Helsinki. Since the study was conducted retrospectively, the Institutional Review Board of Asan Medical Center waived the requirement of informed consent. All methods were performed in accordance with the relevant guidelines and regulations.

## Results

### Characteristics of the study population and comparison of characteristics of patients with IPF and non-IPF ILD

**Fig. 1:**
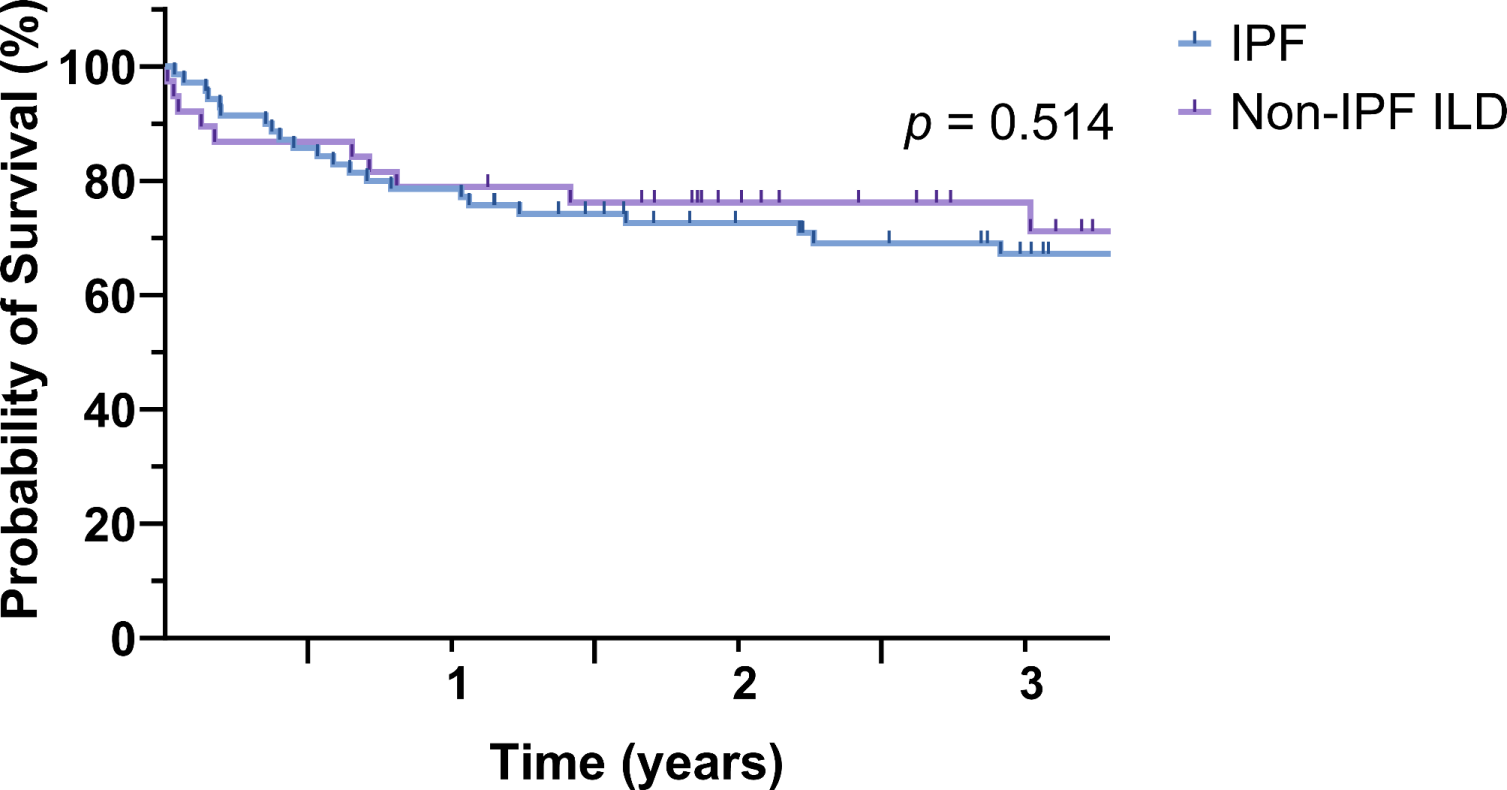
Kaplan–Meier survival analysis for patients with IPF versus non-IPF ILD patients

We reviewed the medical records of 108 patients who underwent bilateral lung transplantation for predisposing ILD. Among them, 77 (71.3%) were male, and the mean age was 57.5 ± 8.2 years. Of the 108 patients, 70 (64.8%) were diagnosed with IPF, while 38 (35.2%) were diagnosed with non-IPF ILD. All patients with non-IPF ILD met the criteria of PPF. Pre-transplant mechanical ventilation was administered to 75% of the patients. Table 1 lists the characteristics and clinical outcomes of patients with IPF and non-IPF ILD. There was no difference in the rate of pre-existing AE between patients with IPF and those with non-IPF ILD (52.9% vs. 39.5%; *p* = 0.184). Patients with IPF were significantly older than those with non-IPF ILD (59.8 vs. 53.3 years; *p* < 0.001), and had a significant predominance of males (81.4% vs. 52.6%; *p* = 0.002) and ever-smokers (65.7% vs. 39.5%; *p* = 0.009). The use of pirfenidone was also significantly more common in patients with IPF (51.4% vs. 2.6%; *p* < 0.001) compared with those with non-IPF ILD. However, the two groups did not differ significantly in other characteristics such as AE status, preoperative steroid use, preoperative mechanical ventilation, and ECMO status. Out of 70 patients with IPF, 37 were diagnosed with AE-IPF, with 32 of them receiving steroids due to these pre-existing AEs. Five patients with AE-IPF did not receive steroids even though they met the AE definition, as there was a high risk of infection, leading to a diagnosis of triggered AE. High-dose steroids were intentionally avoided from the initial stages of clinical management, considering the potential for subsequent transplantation. An additional 25 IPF patients, not diagnosed with pre-existing AE, also received steroids. Among them, 22 were on a tapering regimen due to prior AEs that occurred more than three months earlier. Three patients were prescribed steroids for septic shock. Postoperative variables, including the duration of postoperative ICU stay, also showed no significant differences. The comparison of the survival curves between the two groups revealed no statistically significant difference (Fig. 1).

**Table 1:** Comparison of clinical characteristics of transplant recipients with IPF and with non-IPF ILD

Characteristic	Total	IPF	Non-IPF ILD	*P*-value	
Pre-existing acute exacerbation*	52 (48.1)	37 (52.9)	15 (39.5)	0.184	
Number of recipients	108	70	38		
Mean recipient age, years	57.5 ± 8.2	59.8 ± 6.4	53.3 ± 9.5	< 0.001	
Male sex	77 (71.3)	57 (81.4)	20 (52.6)	0.002	
BMI, kg/m^2^	22.4 ± 3.7	22.5 ± 3.3	22.2 ± 4.3	0.712	
Diabetes mellitus	23 (21.3)	14 (20.0)	9 (23.7)	0.665	
Ever smoker	61 (56.5)	46 (65.7)	15 (39.5)	0.009	
FVC, % predicted	44.1 ± 14.5	44.5 ± 14.2	43.4 ± 15.3	0.750	
DLco, % predicted	24.3 ± 12.6	23.7 ± 11.8	25.5 ± 14.3	0.554	
ILD-GAP index	4.2 ± 1.7	5.1 ± 1.2	2.3 ± 0.9	< 0.001	
Pre-existing pulmonary hypertension	49 (45.4)	31 (44.3)	18 (47.4)	0.759	
Preoperative infection	29 (26.9)	18 (25.7)	11 (28.9)	0.717	
Prior thoracic surgery	46 (42.6)	33 (47.1)	13 (34.2)	0.194	
Pirfenidone use	37 (34.3)	36 (51.4)	1 (2.6)	< 0.001	
Nintedanib use	7 (6.5)	7 (10.0)	0 (0.0)	0.050	
Preoperative steroid use	93 (85.2)	57 (81.4)	36 (94.7)	0.189	
<0.5 mg/kg/day	65 (60.2)	42 (60.0)	23 (60.5)		
0.5–1.0 mg/kg/day	18 (16.7)	10 (14.3)	8 (21.1)		
>1.0 mg/kg/day	10 (9.3)	5 (7.1)	5 (13.2)		
Preoperative MV	81 (75.0)	50 (71.4)	31 (81.6)	0.245	
Duration of preoperative MV (days)	15.3 ± 18.5	14.3 ± 19.2	17.1 ± 17.1	0.473	
Preoperative ECMO	69 (63.9)	41 (58.6)	28 (73.7)	0.118	
Duration of preoperative ECMO (days)	14.6 ± 12.4	13.2 ± 11.3	16.6 ± 13.8	0.270	
Ischemic time (min)	334.2 ± 77.6	330.6 ± 76.5	340.8 ± 80.1	0.518	
Postoperative ECMO	7 (6.5)	3 (4.3)	4 (10.5)	0.239	
Length of postoperative ICU stay (days)	34.1 ± 106.6	24.5 ± 27.9	51.8 ± 175.8	0.348	

Data are presented as means ± standard deviations, or number (%)

*BMI* body mass index, *DLco* diffusing capacity, *ECMO* extracorporeal membrane oxygenation, *FVC* forced vital capacity, *ICU* intensive care unit, *IPF* idiopathic pulmonary fibrosis, *MV* mechanical ventilation, *non-IPF ILD* non–idiopathic pulmonary fibrosis interstitial lung disease

*At the time of lung transplantation

### Comparison of characteristics of patients with and without pre-existing acute exacerbation

A total of 52 (48.1%) patients experienced pre-existing AE-ILD at the time of lung transplantation. Table 2 presents the baseline characteristics of patients with and without AE-ILD. For the most part, there were no significant differences in the baseline characteristics between the two groups; however, a significantly higher proportion of patients with AE-ILD (96.2% versus 55.4%; *p* < 0.001) required preoperative mechanical ventilation during lung transplantation compared with those without AE-ILD. Moreover, the proportion of recipients who underwent preoperative ECMO was also higher among patients with AE-ILD (84.6% vs. 44.6%; *p* < 0.001) than those without AE-ILD. Other characteristics, including mean age of recipients, types of ILD, pulmonary function, and ILD-GAP index, did not differ significantly between the two groups. The duration of postoperative ICU stay did not differ significantly between the two groups (Table 2).

**Table 2:** Comparison of clinical characteristics of transplant recipients with and without pre-existing acute exacerbation at the time of lung transplantation

Characteristic	Total	Acute exacerbation	No acute exacerbation	*P*-value
Number of recipients	108	52	56	
Mean recipient age, years	57.5 ± 8.2	58.2 ± 7.7	56.8 ± 8.6	0.393
Male sex	77 (71.3)	37 (71.2)	40 (71.4)	0.975
BMI, kg/m^2^	22.4 ± 3.7	22.3 ± 3.9	22.5 ± 3.5	0.813
Diagnosis				0.184
IPF	70 (64.8)	37 (71.2)	33 (58.9)	
Non-IPF ILD	38 (35.2)	15 (28.8)	23 (41.1)	
CTD-ILD/IPAF	19 (17.6)	6 (11.5)	13 (23.2)	
SSc-ILD	4 (3.7)	1 (1.9)	3 (5.4)	
SJS-ILD	4 (3.7)	2 (3.8)	2 (3.6)	
IIM-ILD	5 (4.6)	1 (1.9)	4 (7.1)	
Other CTD-ILD/IPAF	6 (5.6)	2 (3.8)	4 (7.1)	
Idiopathic NSIP	6 (5.6)	5 (9.6)	1 (1.8)	
Chronic HP	2 (1.9)	0	2 (3.6)	
Unclassifiable ILD	11 (6.5)	7 (13.5)	7 (12.5)	
Diabetes mellitus	23 (21.3)	7 (13.5)	16 (28.6)	0.055
Ever smoker	61 (56.5)	33 (63.5)	28 (50.0)	0.159
FVC, % predicted	44.1 ± 14.5	44.1 ± 16.4	44.1 ± 12.9	0.987
DLco, % predicted	24.3 ± 12.6	26.1 ± 12.1	22.7 ± 12.9	0.227
ILD-GAP index	4.2 ± 1.7	4.4 ± 1.9	4.0 ± 1.6	0.280
Pre-existing pulmonary hypertension	49 (45.4)	23 (44.2)	26 (46.4)	0.819
Preoperative infection	29 (26.9)	17 (32.7)	12 (21.4)	0.187
Prior thoracic surgery	46 (42.6)	21 (40.4)	25 (44.6)	0.655
Pirfenidone use	37 (34.3)	19 (36.5)	18 (32.1)	0.631
Nintedanib use	7 (6.5)	4 (7.7)	3 (5.4)	0.709
Preoperative steroid use	93 (86.1)	47 (90.4)	46 (82.1)	0.216
<0.5 mg/kg/day	65 (60.2)	32 (61.5)	33 (58.9)	
0.5–1.0 mg/kg/day	18 (16.7)	9 (17.3)	9 (16.1)	
>1.0 mg/kg/day	10 (9.3)	6 (11.5)	4 (7.1)	
Preoperative MV	81 (75.0)	50 (96.2)	31 (55.4)	< 0.001
Duration of preoperative MV (days)	15.3 ± 18.5	16.6 ± 12.3	13.9 ± 23.8	0.488
Preoperative ECMO	69 (63.9)	44 (84.6)	25 (44.6)	< 0.001
Duration of preoperative ECMO (days)	14.6 ± 12.4	14.6 ± 12.4	14.6 ± 12.7	0.998
Ischemic time (min)	334.2 ± 77.6	331.7 ± 81.0	336.5 ± 74.9	0.750
Postoperative ECMO	7 (6.5)	5 (9.6)	2 (3.6)	0.258
Length of postoperative ICU stay (days)	34.1 ± 106.6	28.0 ± 29.4	39.8 ± 145.7	0.569
Mean follow-up (years)	3.2 ± 2.6	3.0 ± 2.7	3.3 ± 2.6	0.439
90-day survival, %	89.9	86.5	92.9	
1-year survival, %	78.7	73.1	83.9	
3-year survival, %	70.1	60.1	79.6	

Data are presented as means ± standard deviations, or number (%)

*BMI* body mass index, *COP* cryptogenic organizing pneumonia, *CTD* connective tissue disease, *DLco* diffusing capacity, *ECMO* extracorporeal membrane oxygenation, *FVC* forced vital capacity, *HP* hypersensitivity pneumonitis, *ICU* intensive care unit, *IIM* idiopathic inflammatory myopathies, *ILD* interstitial lung disease, *IPAF* interstitial pneumonia with autoimmune features, *IPF* idiopathic pulmonary fibrosis, *MV* mechanical ventilation, *non-IPF* non–idiopathic pulmonary fibrosis, *NSIP* non-specific interstitial pneumonia, *SJS* Sjögren’s syndrome, *SSc* systemic sclerosis

### Effect of pre-existing acute exacerbation on survival after lung transplantation

**Fig. 2:**
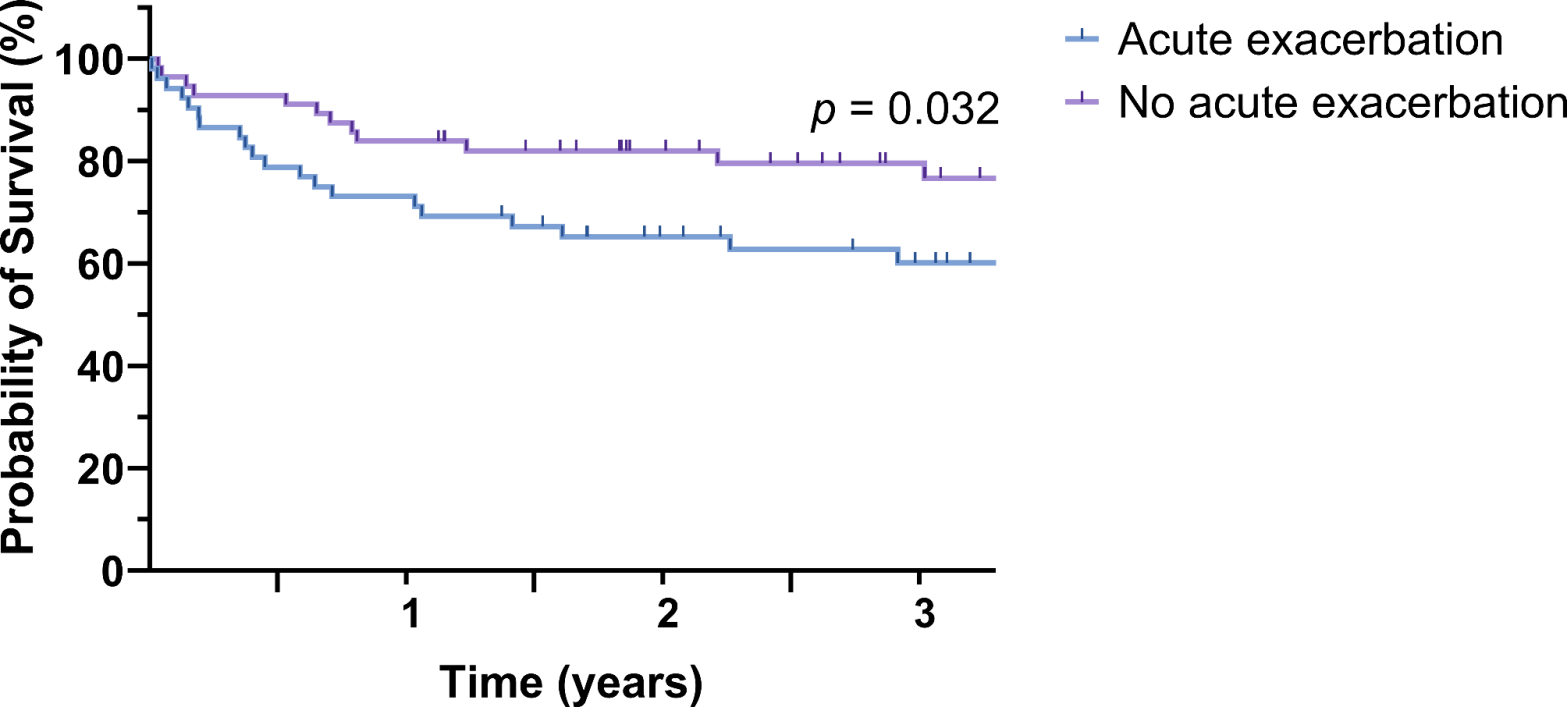
Kaplan–Meier survival analysis for patients with and without pre-existing acute exacerbation

In the study population, the cumulative mortality at 90-day, 1-year, and 3-year were 10.1%, 21.3%, and 29.9%, respectively. Thirty-six patients (33.3%) died during the study period, with 23 of these patients having AE-ILD and 13 without AE-ILD. (Table 3) Infection was the leading cause of death among these patients, accounting for 61.1% (22/36) of deaths, followed by chronic lung allograft rejection and postoperative complications, each at 8.3% (3/36). The mean follow-up duration did not significantly differ between the two groups, with values of 3.0 ± 2.7 years and 3.3 ± 2.6 years for patients with and without AE, respectively (*p =* 0.439). The cumulative mortality of 90 days, 1 year, and 3 years were 13.5%, 26.9%, and 39.9% for patients with AE, and 7.1%, 16.1%, and 20.4% for patients without AE, respectively (Table 2). Figure 2 compares the survival curves between the two groups, revealing significantly higher mortality rates in patients with AE than those without AE (*p =* 0.032). Additionally, the survival curves classified according to the time of lung transplantation did not show statistically significant differences (Additional file 1).

**Table 3:** Comparison of characteristics and clinical outcomes in transplant recipients who did and did not survive

Characteristic	Total	Patients who survived	Patients who did not survive	*P*-value
Number of recipients	108	72	36	
Mean recipient age, years	57.5 ± 8.2	56.7 ± 8.4	59.0 ± 7.7	0.169
Male sex	77 (71.3)	50 (69.4)	27 (75.0)	0.547
BMI, kg/m^2^	22.4 ± 3.7	22.2 ± 3.4	22.9 ± 4.1	0.336
Diagnosis				0.254
IPF	70 (64.8)	44 (61.1)	26 (72.2)	
Non-IPF ILD	38 (35.2)	28 (38.9)	10 (27.8)	
CTD-ILD/IPAF	19 (17.6)	10 (13.9)	9 (25.0)	
SSc-ILD	4 (3.7)	4 (5.6)	0	
SJS-ILD	4 (3.7)	2 (2.8)	2 (5.6)	
IIM-ILD	5 (4.6)	2 (2.8)	3 (8.3)	
Other CTD-ILD/IPAF	6 (5.6)	2 (2.8)	4 (11.1)	
Idiopathic NSIP	6 (5.6)	6 (8.3)	0 (0.0)	
Chronic HP	2 (1.9)	2 (2.8)	0	
Unclassifiable ILD	11 (6.5)	10 (13.9)	1 (2.8)	
Diabetes mellitus	23 (21.3)	15 (20.8)	8 (22.2)	0.868
Ever smoker	61 (56.5)	40 (55.6)	21 (58.3)	0.784
FVC, % predicted	44.1 ± 14.5	42.9 ± 12.1	44.8 ± 15.7	0.570
DLco, % predicted	24.3 ± 12.6	24.3 ± 12.2	24.3 ± 12.9	0.999
ILD-GAP index	4.2 ± 1.7	4.4 ± 2.0	4.1 ± 1.6	0.487
Pre-existing pulmonary hypertension	49 (45.4)	30 (41.7)	19 (52.8)	0.274
Preoperative infection	29 (26.9)	19 (26.4)	10 (27.8)	0.878
Pre-existing acute exacerbation	52 (48.1)	29 (40.3)	23 (63.9)	0.021
Prior thoracic surgery	46 (42.6)	30 (41.7)	16 (44.4)	0.783
Pirfenidone use	37 (34.3)	26 (36.1)	11 (30.6)	0.566
Nintedanib use	7 (6.5)	3 (4.2)	4 (11.1)	0.219
Preoperative steroid use	93 (86.1)	59 (81.9)	34 (94.4)	0.077
<0.5 mg/kg/day	65 (60.2)	41 (56.9)	24 (66.7)	
0.5–1.0 mg/kg/day	18 (16.7)	11 (15.3)	7 (19.4)	
>1.0 mg/kg/day	10 (9.3)	7 (9.7)	3 (8.3)	
Preoperative MV	81 (75.0)	53 (73.6)	28 (77.8)	0.637
Duration of preoperative MV (days)	15.3 ± 18.5	14.7 ± 19.1	16.6 ± 17.4	0.635
Preoperative ECMO	69 (63.9)	46 (63.9)	23 (63.9)	> 0.999
Duration of preoperative ECMO (days)	14.6 ± 12.4	13.5 ± 11.2	16.8 ± 14.6	0.297
Ischemic time (min)	334.2 ± 77.6	335.8 ± 81.3	330.9 ± 70.3	0.763
Postoperative ECMO	7 (6.5)	6 (8.3)	1 (2.8)	0.420
Length of postoperative ICU stay (days)	34.1 ± 106.6	21.8 ± 19.3	58.8 ± 181.7	0.230
Mean follow-up (years)	3.2 ± 2.6	4.1 ± 2.5	1.3 ± 2.0	< 0.001

Data are presented as means ± standard deviations, or number (%)

*BMI* body mass index, *COP* cryptogenic organizing pneumonia, *CTD* connective tissue disease, *DLco* diffusing capacity, *ECMO* extracorporeal membrane oxygenation, *FVC* forced vital capacity, *HP* hypersensitivity pneumonitis, *ICU* intensive care unit, *IIM* idiopathic inflammatory myopathies, *ILD* interstitial lung disease, *IPAF* interstitial pneumonia with autoimmune features, *IPF* idiopathic pulmonary fibrosis, *MV* mechanical ventilation, *non-IPF* non–idiopathic pulmonary fibrosis, *NSIP* non-specific interstitial pneumonia, *SJS* Sjögren’s syndrome, *SSc* systemic sclerosis

### Factors influencing mortality in the study population

The univariate Cox analysis revealed that pre-existing AE (hazard ratio [HR], 2.072; *p* = 0.036) in transplant recipients was significantly associated with higher mortality. The association between preoperative steroid use and mortality was marginally significant (HR, 3.610; *p* = 0.078) (Table 4). The multivariate Cox analysis demonstrated that in transplant recipients, pre-existing AE (HR, 2.078; *p* = 0.041) remained a significant independent risk factor for mortality after adjusting for the age of the recipient (HR, 1.048; *p* = 0.078), preoperative steroid use (HR, 3.976; *p* = 0.064), and length of postoperative ICU stay (HR, 1.001; *p* = 0.124). When focusing on 3-year mortality, we identified both pre-existing AE and recipient age as marginally significant prognostic factors (Additional file 2).

**Table 4:** Risk factors for mortality according to Cox proportional hazards model

Parameter	Hazard ratio	95% confidence interval	*P*-value
Univariable analysis			
Recipient age, years	1.036	0.988–1.087	0.148
Male sex	1.119	0.524–2.391	0.771
BMI, kg/m^2^	1.037	0.946–1.136	0.440
Diabetes mellitus	0.988	0.450–2.170	0.976
Ever smoker	0.999	0.511–1.953	0.997
FVC, % predicted	0.991	0.965–1.018	0.500
DLco, % predicted	1.003	0.973–1.033	0.870
ILD-GAP index	1.067	0.855–1.332	0.567
Pre-existing pulmonary hypertension	1.281	0.660–2.487	0.464
Preoperative infection	0.990	0.476–2.060	0.979
IPF versus non-IPF ILD	1.277	0.612–2.662	0.515
Pre-existing acute exacerbation	2.072	1.048–4.097	0.036
Pirfenidone use	0.925	0.453–1.891	0.831
Nintedanib use	1.814	0.640–5.145	0.263
Preoperative steroid use	3.610	0.864–15.086	0.078
Preoperative immunosuppressant use	1.659	0.722–3.815	0.233
Preoperative MV	1.191	0.541–2.624	0.664
Duration of preoperative MV (days)	1.005	0.990–1.020	0.532
Preoperative ECMO	1.117	0.563–2.215	0.751
Duration of preoperative ECMO (days)	1.017	0.986–1.049	0.281
Postoperative ECMO	0.442	0.060–3.237	0.422
Length of postoperative ICU stay (days)	1.001	0.999–1.003	0.187
Prior thoracic surgery	1.137	0.589–2.196	0.702
Multivariable analysis			
Recipient age, years	1.048	0.995–1.104	0.078
Pre-existing acute exacerbation	2.078	1.031–4.188	0.041
Preoperative steroid use	3.976	0.925–17.085	0.064
Length of postoperative ICU stay (days)	1.001	1.000–1.003	0.124

*BMI* body mass index, *DLco* diffusing capacity, *ECMO* extracorporeal membrane oxygenation, *FVC* forced vital capacity, *ICU* intensive care unit, *MV* mechanical ventilation

## Discussion

In this study, 52 (48.1%) patients experienced AE-ILD at the time of lung transplantation. Although the type of ILD (IPF vs. non-IPF ILD) did not affect clinical outcomes after lung transplantation, the AE-ILD group showed worse overall survival than the non-AE-ILD group. In addition, pre-existing AE was an independent prognostic factor for mortality in patients with ILD who underwent lung transplantation.

The lung allocation system in South Korea is primarily based on medical urgency, which differs from other countries [17, 18]. Therefore, patients with status 0, meaning patients receiving mechanical ventilation or ECMO, are regarded as the highest priority for transplantation in Korea. Due to a donor shortage, 68.6% of transplant patients were status 0, and 38.5% underwent ECMO as a bridge to transplantation in 2019 [15]. In our study, 75.0% of patients received preoperative mechanical ventilation and 63.9% underwent ECMO as a bridge to transplantation.

AE-IPF has been characterized by acute, clinically significant respiratory deterioration [7]. The etiology of AE remains to be fully established, but several known risk factors and triggers include advanced disease, infection, and a previous history of AE [8, 19, 20]. According to previous studies, there was a weighted average of 41 AE of 1000 patient-years in patients with IPF [21]. The impact of an AE-IPF on prognosis is significant, and the median survival in AE-IPF was approximately 3 to 4 months [8, 22]. Within a clinical course of ILD other than IPF, the presentation of AE is similar to IPF, and AE is associated with significant morbidity and mortality in non-IPF ILD [10, 23].

Although lung transplantation could be considered a life-extending treatment option for patients with advanced ILD [24], the clinical impact of pre-transplant AE showed conflicting results. Chizinga et al., in 25 patients with AE-ILD and 67 patients with stable ILD who underwent lung transplantation, reported that survival at 1 year for the transplanted patients did not differ between the two groups (96% for AE-ILD group vs. 92.5% for stable ILD group) [11]. However, Dotan et al. showed that patients in the AE-IPF group (n = 28) had significantly worse survival compared with patients transplanted during stable IPF (n = 52) (survival rates at 1, 2, and 3 years of 71%, 60%, and 60% vs. 94%, 90%, and 90%, respectively) [12]. These conflicting results may be attributed to the patient characteristics at the time of lung transplantation in the AE-ILD group. Although a total of 25% of AE-IPF patients (7/28 patients) received ventilatory support (mechanical ventilator or ECMO) in Dotan’s study, only 1 (4%) patient in the AE-ILD group received ECMO in Chizinga’s study, suggesting that the severity of the condition of the patients with AE included in the two studies differed. Our current study (75% of patients received mechanical ventilation) also showed that the AE-ILD group had worse survival than the non-AE-ILD group. These findings suggest that lung transplantation should be performed on selective patients, especially in patients with severe acute exacerbation requiring ventilatory support.

While the proportion of pre-operative mechanical ventilation was high in the overall study population, there was a significant difference in the pre-operative mechanical ventilation rates between ILD patients with and without pre-existing AE (96.2% vs. 55.4%; *p* < 0.001). Although we could not verify the performance status or rehabilitation capacity of transplant recipients at the time of transplantation, the high rate of mechanical ventilation in patients with pre-existing AE suggests a poor performance status associated with a complicated clinical course with high severity. These differences might have contributed to the higher mortality rate observed.

Several clinical parameters have been suggested as prognostic factors after lung transplantation, including pre-operative diagnosis, type of surgical procedure, and donor/recipient characteristics [25, 26]. In our current study, the IPF group showed a similar prognosis to the non-IPF ILD group after lung transplantation, comparable with previous studies [24, 27]. In addition, despite a lack of statistical significance, old recipient age, and preoperative steroid usage were associated with mortality in our study, and similar findings were reported in previous studies [28–31]. Several studies have reported inconsistent lung transplantation outcomes in patients who received preoperative ventilation and ECMO [32–34]. In our current study, while pre-existing AE was an independent prognostic factor, pre-transplant mechanical respiratory support was not significantly associated with prognosis. Although defining the reason for these results remains challenging, there are possible explanations. Approximately half of the patients without pre-existing AE required preoperative mechanical ventilation and ECMO due to clinical deterioration caused by conditions such as pneumonia, pulmonary thromboembolism, and pneumothorax. It is conceivable that the immune responses linked to AE might have exerted an influence. AE is characterized by an aberrant immune response and increased lung inflammation [35–37]. This immune dysregulation could persist even after lung transplantation, possibly leading to post-transplant complications and poorer outcomes in patients with AE-ILD. Further studies are warranted to confirm these findings.

This study had several limitations. First, it was a retrospective single-center study. Additionally, the lung allocation system in South Korea is primarily based on urgency, resulting in a high frequency of AE, mechanical ventilation and ECMO applications among the recipients in this study. These aspects could potentially limit the generalizability of our findings. Second, it was difficult to analyze the clinical impact of lung transplantation in ILD patients with AE because the patients who did not receive lung transplantation were not included in the current analysis. Third, some of the non-AE-ILD patients had experienced previous AE events and were undergoing a steroid tapering regimen at the time of lung transplantation, making it challenging to categorize them as purely stable ILD. However, these prior AE events occurred more than three months earlier, and the patients showed clinical and radiological improvement. Lastly, including potential confounders such as general condition or rehabilitation capacity in the analysis was challenging, and might have influenced mortality outcomes.

In conclusion, AE-ILD was an independent prognostic factor for mortality in patients who received lung transplantation. Although lung transplantation remains an effective treatment option for ILD patients with pre-existing AE, our results suggest that caution is required during decision-making, especially in patients requiring pre-transplant mechanical respiratory support.

### Electronic supplementary material

Below is the link to the electronic supplementary material.

Supplementary Material 1

Supplementary Material 2

## Data Availability

The datasets generated or analyzed during the current study are available from the corresponding author upon reasonable request.
