# Supplementary material for: Clinical impact of pre-existing acute exacerbation in patients with interstitial lung disease who underwent lung transplantation
Source: Respir Res. 2023 Dec 7;24:307. doi: 10.1186/s12931-023-02614-z (PMC10701919; doi:10.1186/s12931-023-02614-z)
Supplement: Supplementary file 1 — Supplementary Material 1 [file 12931_2023_2614_MOESM1_ESM.docx]

**Additional file 2**

Table S1. Comparison of characteristics and clinical outcomes in transplant recipients who did and did not survive three years post-operation.

| Characteristic | Total | Patients who survived | Patients who did not survive | *P*-value |
| --- | --- | --- | --- | --- |
| Number of recipients | 108 | 77 | 31 |  |
| Mean recipient age, years | 57.5 ± 8.2 | 56.7 ± 8.2 | 59.4 ± 8.0 | 0.134 |
| Male sex | 77 (71.3) | 54 (70.1) | 23 (74.2) | 0.673 |
| BMI, kg/m^2^ | 22.4 ± 3.7 | 22.3 ± 3.4 | 22.7 ± 4.4 | 0.604 |
| Diagnosis |  | | | 0.396 |
| IPF | 70 (64.8) | 48 (62.3) | 22 (71.0) |  |
| Non-IPF ILD | 38 (35.2) | 29 (37.7) | 9 (29.0) |  |
| CTD-ILD/IPAF | 19 (17.6) | 11 (14.3) | 8 (25.8) |  |
| SSc-ILD | 4 (3.7) | 4 (5.2) | 0 |  |
| SJS-ILD | 4 (3.7) | 2 (2.6) | 2 (6.5) |  |
| IIM-ILD | 5 (4.6) | 3 (3.9) | 2 (6.5) |  |
| Other CTD-ILD/IPAF | 6 (5.6) | 2 (2.6) | 4 (12.9) |  |
| Idiopathic NSIP | 6 (5.6) | 6 (7.8) | 0 |  |
| Chronic HP | 2 (1.9) | 1 (1.3) | 1 (3.2) |  |
| Unclassifiable ILD | 11 (6.5) | 11 (14.3) | 0 |  |
| Diabetes mellitus | 23 (21.3) | 16 (20.8) | 7 (22.6) | 0.836 |
| Ever smoker | 61 (56.5) | 43 (55.8) | 18 (58.1) | 0.833 |
| FVC, % predicted | 44.1 ± 14.5 | 44.9 ± 15.2 | 42.3 ± 12.7 | 0.465 |
| DLco, % predicted | 24.3 ± 12.6 | 24.2 ± 12.7 | 24.5 ± 12.6 | 0.924 |
| ILD-GAP index | 4.2 ± 1.7 | 4.1 ± 1.6 | 4.5 ± 2.0 | 0.333 |
| Preexisting pulmonary hypertension | 49 (45.4) | 33 (42.9) | 16 (51.6) | 0.408 |
| Preoperative infection | 29 (26.9) | 20 (26.0) | 9 (29.0) | 0.746 |
| Acute exacerbation | 52 (48.1) | 32 (41.6) | 20 (64.5) | 0.031 |
| Prior thoracic surgery | 46 (42.6) | 32 (41.6) | 14 (45.2) | 0.732 |
| Pirfenidone use | 37 (34.3) | 27 (35.1) | 10 (32.3) | 0.781 |
| Nintedanib use | 7 (6.5) | 4 (5.2) | 3 (9.7) | 0.407 |
| Preoperative steroid use | 93 (86.1) | 64 (83.1) | 29 (93.5) | 0.223 |
| <0.5 mg/kg/day | 65 (60.2) | 45 (58.4) | 20 (64.5) |  |
| 0.5–1.0 mg/kg/day | 18 (16.7) | 12 (15.6) | 6 (19.4) |  |
| >1.0 mg/kg/day | 10 (9.3) | 7 (9.1) | 3 (9.7) |  |
| Preoperative MV | 81 (75.0) | 58 (75.3) | 23 (74.2) | 0.902 |
| Duration of preoperative MV (days) | 15.3 ± 18.5 | 14.7 ± 18.5 | 16.9 ± 18.6 | 0.599 |
| Preoperative ECMO | 69 (63.9) | 50 (64.9) | 19 (61.3) | 0.721 |
| Duration of preoperative ECMO (days) | 14.6 ± 12.4 | 13.3 ± 11.1 | 17.8 ± 15.2 | 0.178 |
| Ischemic time (min) | 334.2 ± 77.6 | 81.3 ± 9.3 | 68.4 ± 12.5 | 0.818 |
| Postoperative ECMO | 7 (6.5) | 6 (7.8) | 1 (3.2) | 0.671 |
| Length of postoperative ICU stay (days) | 34.1 ± 106.6 | 35.3 ± 124.6 | 31.1 ± 34.2 | 0.855 |
| Mean follow-up (years) | 3.2 ± 2.6 | 4.1 ± 2.5 | 0.7 ± 0.7 | <0.001 |

Data are presented as means ± standard deviations, or number (%).

*BMI* body mass index, *COP* cryptogenic organizing pneumonia, *CTD* connective tissue disease, *ECMO* extracorporeal membrane oxygenation, *HP* hypersensitivity pneumonitis, *ICU* intensive care unit, *IIM* idiopathic inflammatory myopathies, *ILD* interstitial lung disease, *IPAF* interstitial pneumonia with autoimmune features, *IPF* idiopathic pulmonary fibrosis, *MV* mechanical ventilation, *non-IPF* non–idiopathic pulmonary fibrosis, *NSIP* non-specific interstitial pneumonia, *SJS* Sjögren’s syndrome, *SSc* systemic sclerosis.

Table S2. Risk factors for 3-year mortality according to Cox proportional hazards model

| Parameter | Hazard ratio | 95% confidence interval | *P*-value |
| --- | --- | --- | --- |
| Univariable analysis | | | |
| Recipient age, years | 1.037 | 0.985–1.091 | 0.167 |
| Male sex | 1.154 | 0.516–2.581 | 0.728 |
| BMI, kg/m^2^ | 1.026 | 0.931–1.129 | 0.608 |
| Diabetes mellitus | 1.036 | 0.446–2.406 | 0.934 |
| Ever smoker | 1.067 | 0.523–2.178 | 0.859 |
| FVC, % predicted | 0.988 | 0.959–1.017 | 0.412 |
| DLco, % predicted | 1.002 | 0.971–1.034 | 0.897 |
| ILD-GAP index | 1.106 | 0.873–1.401 | 0.404 |
| Preexisting pulmonary hypertension | 1.316 | 0.650–2.662 | 0.445 |
| Preoperative infection | 1.128 | 0.519–2.454 | 0.761 |
| IPF versus non-IPF ILD | 1.298 | 0.598–2.821 | 0.509 |
| Acute exacerbation | 2.167 | 1.038–4.524 | 0.039 |
| Pirfenidone use | 0.929 | 0.437–1.974 | 0.849 |
| Nintedanib use | 1.449 | 0.440–4.767 | 0.542 |
| Preoperative steroid use | 2.786 | 0.663–11.696 | 0.162 |
| Preoperative immunosuppressant use | 2.026 | 0.871–4.708 | 0.101 |
| Preoperative MV | 0.992 | 0.444–2.218 | 0.984 |
| Duration of preoperative MV (days) | 1.005 | 0.989–1.022 | 0.521 |
| Preoperative ECMO | 0.934 | 0.453–1.925 | 0.854 |
| Postoperative ECMO | 0.473 | 0.064–3.466 | 0.461 |
| Length of postoperative ICU stay (days) | 1.000 | 0.996–1.003 | 0.866 |
| Prior thoracic surgery | 1.151 | 0.567–2.335 | 0.698 |
| Multivariable analysis | | | |
| Recipient age, years | 1.047 | 0.992–1.105 | 0.099 |
| Acute exacerbation | 1.935 | 0.921–4.068 | 0.082 |
| Preoperative steroid use | 2.783 | 0.650–11.908 | 0.168 |
| Preoperative immunosuppressant use | 2.044 | 0.872–4.791 | 0.100 |

*BMI* body mass index, *ECMO* extracorporeal membrane oxygenation, *ICU* intensive care unit, *MV* mechanical ventilation.
