# Supplementary material for: Clinical impact of pre-existing acute exacerbation in patients with interstitial lung disease who underwent lung transplantation
Source: Respir Res. 2023 Dec 7;24:307. doi: 10.1186/s12931-023-02614-z (PMC10701919; doi:10.1186/s12931-023-02614-z)
Supplement: Supplementary file 2 — Supplementary Material 2 [file 12931_2023_2614_MOESM2_ESM.docx]

**Additional file 1.**

Figure S1. Comparison of survival curves based on the time of lung transplantation (A) 2008-2015 vs. 2016-2022 (B) Three-period Comparison: 2008-2015, 2016-2019, 2020-2022


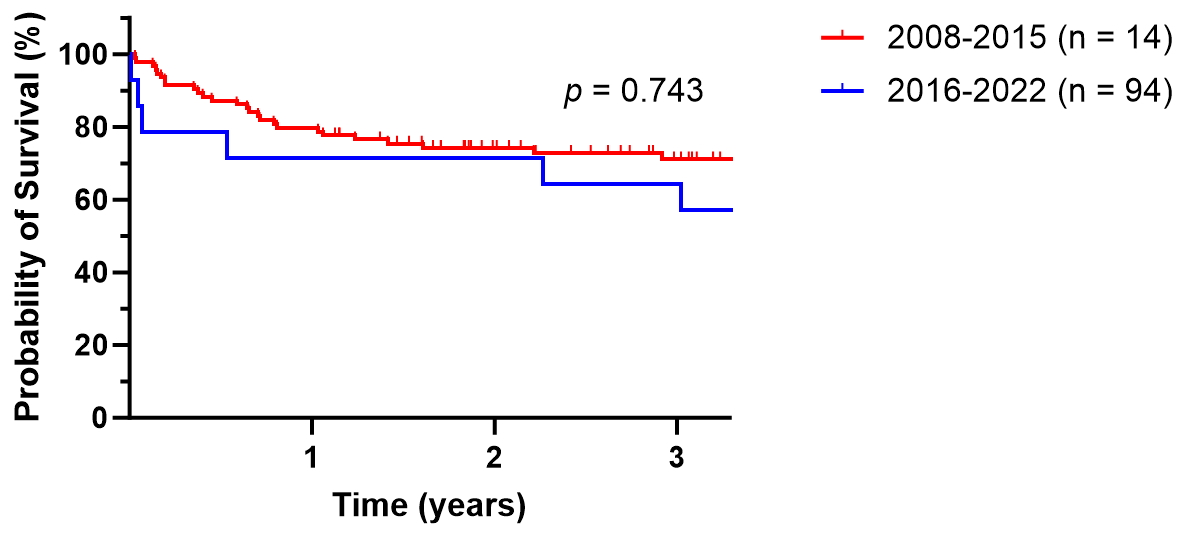
(A)


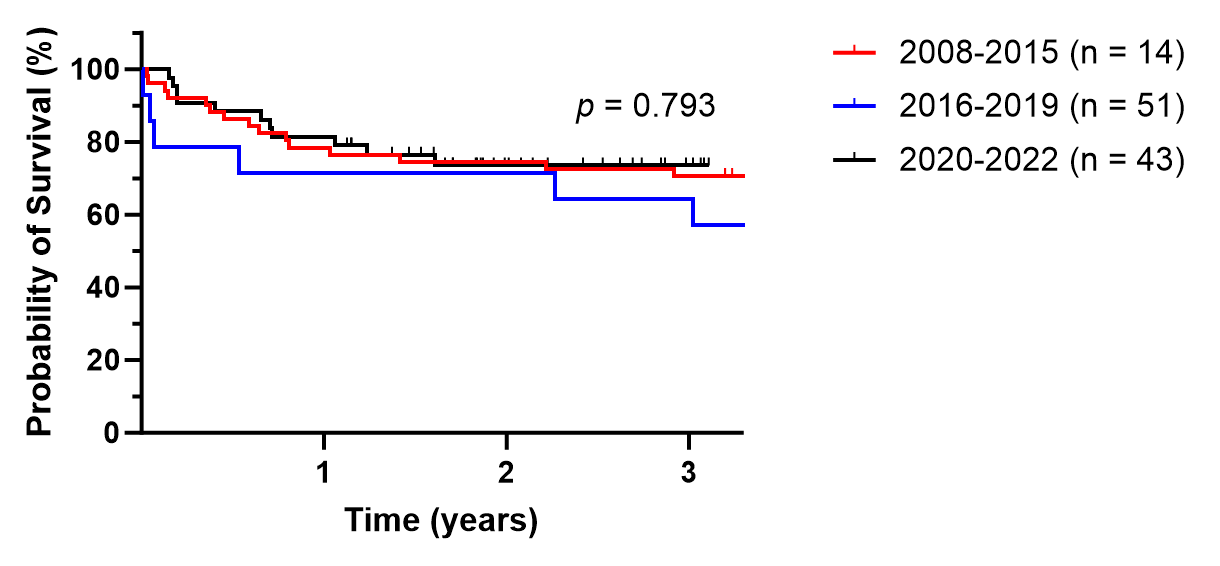
(B)
